# Comment on Skrebinska et al. Who Could Be Blamed in the Case of Discrepant Histology and Serology Results for *Helicobacter pylori* Detection? *Diagnostics* 2022, *12*, 133

**DOI:** 10.3390/diagnostics12061424

**Published:** 2022-06-09

**Authors:** Éva Kocsmár, Gábor Lotz

**Affiliations:** Department of Pathology, Forensic and Insurance Medicine, Semmelweis University, Üllői Street 93, H-1091 Budapest, Hungary; kocsmar.eva@med.semmelweis-univ.hu

**Keywords:** *Helicobacter pylori*, Giemsa staining, immunohistochemistry, active gastritis, diagnostic sensitivity

## Abstract

In their article, Skebrinska and colleagues analysed the potential pitfalls of detecting *Helicobacter pylori* (*H. pylori*) by serology, histological (Giemsa) and immunohistochemical (IHC) staining. However, in the Introduction, the authors state: “…IHC is recommended only in individuals with active gastritis without *H. pylori* identification by histochemistry”. Although this is a widely-held view, it does not seem to hold up in view of the results of the study by Kocsmár et al., which showed that the diagnostic sensitivity of Giemsa in the absence of activity is only 33.6%, but it is 92.6% in the presence of active gastritis, which is close to the 99.4% sensitivity of IHC. Considering that chronic active gastritis with the features of *H. pylori* gastritis is also common in other entities, if active inflammation is present in the sample, there is a very small chance that a Giemsa-negative case will be confirmed as *H. pylori*-positive by IHC. Based on this, the use of IHC is more reasonable in Giemsa-negative cases with no activity in which the etiological role of *H. pylori* is suggested by clinical, anamnestic or other data. However, it may also be reasonable to routinely use IHC as the primary staining method instead of Giemsa.

We have read with great interest the excellent and comprehensive study by Skebrinska and colleagues on the potential pitfalls of detecting *Helicobacter pylori* (*H. pylori*) by serology, histological (Giemsa) and immunohistochemical (IHC) staining, as well as by molecular biological (polymerase chain reaction/PCR/) methods [1]. The conclusions of the study are consistent with what we know about the diagnostic accuracy of each detection technique, yet are forward-looking, clear and practical. We fully agree with the statement in the Results and Discussion section that the results of the study show that tissue-based testing by an experienced pathologist gives more reliable results than serology.

However, in the Introduction of the article, the authors state: “…IHC is recommended only in individuals with active gastritis without *H. pylori* identification by histochemistry”. Although this is a widely held view, it does not seem to hold up in view of the results of a previous study of our group. This study by Kocsmár et al. showed that the sensitivity of Giemsa staining is highly dependent on the presence of activity (intraepithelial neutrophil infiltration) [2]. Accordingly, the diagnostic sensitivity of Giemsa in the absence of activity is only 33.6%, but 92.6% in the presence of active gastritis, which is quite close to the 99.4% sensitivity of IHC. This implies that if active inflammation is present in the sample, there is a very small chance that a Giemsa-negative case will be confirmed as *H. pylori* positive by IHC. In this context, it is important to note that chronic active gastritis with the features of *H. pylori* gastritis is also frequently seen in other entities such as non-steroidal anti-inflammatory drug-induced gastritis, autoimmune gastritis, Crohn disease-associated gastritis or cytomegalovirus-associated gastritis [3,4]. Based on our results, the use of IHC is more reasonable in Giemsa-negative cases with no activity in which the etiological role of *H. pylori* is suggested by clinical, anamnestic or other data. On the other hand, we also understand the need to perform IHC to detect the approximately 6% plus of cases with *H. pylori* infection in Giemsa-negative, active gastritis. However, at this point, it might be reasonably considered that IHC should be routinely used as the primary staining instead of Giemsa [2].

In addition, we would like to reflect on the finding of this study that 6.4% of *H. pylori* serology positive individuals are not *H. pylori* infected, despite having received no previous eradication treatment. This is explained (correctly) by the authors as due to unintended *H. pylori* eradication with antibiotics for another disease. This possibility is supported by a further study by us, which for the first time described the population dynamics of *H. pylori* clarithromycin resistance using mathematical modelling [5]. In the cohort of this study, 1731 of 4744 *H. pylori* infected individuals (36.5%) had a history of prior non-eradication macrolide treatment. This extensive macrolide use may inevitably, albeit relatively infrequently, lead to unintended eradication, resulting in positive *H. pylori* serology in actually uninfected individuals.

Returning to the issues of Giemsa staining and the use of IHC, we consider the conclusions of this study important and recommend PCR testing in all cases where an etiological role of *H. pylori* is clinically suggested but histopathological confirmation of *H. pylori* is not possible, either by conventional or immunohistochemical staining.
